# Rabies in the African Civet: An Incidental Host for Lyssaviruses?

**DOI:** 10.3390/v12040368

**Published:** 2020-03-27

**Authors:** Claude T. Sabeta, Denise A. Marston, Lorraine M. McElhinney, Daniel L. Horton, Baby M. N. Phahladira, Anthony R. Fooks

**Affiliations:** 1 Agricultural Research Council, Onderstepoort Veterinary Institute, OIE Rabies Reference Laboratory, Pretoria 0110, South Africa; PhahladiraB@arc.agric.za; 2 Department of Veterinary Tropical Diseases, University of Pretoria, Faculty of Veterinary Sciences, Onderstepoort, Pretoria 0110, South Africa; 3 OIE Rabies Reference Laboratory, Wildlife Zoonoses and Vector Borne Diseases Research Group, Animal and Plant Health Agency (APHA, Weybridge), Surrey KT15 3NB, UK; Denise.Marston@apha.gov.uk (D.A.M.); Lorraine.McElhinney@apha.gov.uk (L.M.M.); Tony.Fooks@apha.gov.uk (A.R.F.); 4 Institute of Infection and Global Health, University of Liverpool, Liverpool L7 3EA, UK; 5 School of Veterinary Medicine, University of Surrey, Guildford GU2 7XH, UK; d.horton@surrey.ac.uk; 6 Faculty of Health and Life Sciences, University of Liverpool, Liverpool L69 3GB, UK; 7 Institute for Infection and Immunity, St. George’s University of London, London SW17 0RE, UK

**Keywords:** rabies virus, African civet, incidental host

## Abstract

In South Africa, canid rabies virus (RABV) infection is maintained in domestic and wildlife species. The identification of rabies in African civets raised the question of whether this wildlife carnivore is a potential reservoir host of RABVs of direct and ancestral dog origin (dog-maintained and dog-derived origins) with an independent cycle of transmission. Genetic analyses of African civet nucleoprotein sequences for 23 African civet RABVs and historically published sequences demonstrated that RABVs from African civets have two origins related to dog and mongoose rabies enzootics. The data support observations of the interaction of civets with domestic dogs and wildlife mongooses, mostly in Northern South Africa and North-East Zimbabwe. Within each host species clade, African civet RABVs group exclusively together, implying intra-species virus transfer occurs readily. The canid RABV clade appears to support virus transfer more readily between hosts than mongoose RABVs. Furthermore, these data probably indicate short transmission chains with conspecifics that may be related to transient rabies maintenance in African civets. Hence, it is important to continue monitoring the emergence of lyssaviruses in this host. Observations from this study are supported by ongoing and independent similar cases, in which bat-eared foxes and black-backed jackal species maintain independent rabies cycles of what were once dog-maintained RABVs.

## 1. Introduction

Lyssaviruses are highly neurotropic viruses characterised by non-segmented and negative-stranded RNA genomes. These viruses are typically bullet-shaped and belong to the *Rhabdoviridae* family and the *Lyssavirus* genus [1,2]. The viral genome is approximately 12 kb in size and encodes information for five proteins, namely the nucleoprotein (N), the matrix (M) protein, the phosphoprotein (P), the glycoprotein (G) and the RNA-dependent polymerase (L) [1,2].

All members of the genus *Lyssavirus* are aetiologic agents of rabies, and are capable of inducing a fatal encephalitic disease in susceptible host species, including humans, a disease now considered neglected throughout many regions of the world [3,4] particularly in Africa and Asia. Rabies remains an important disease from both a veterinary and public health standpoint and at least 59,000 human deaths occur due to the disease every year [5,6,7]. The domestic dog is the principal source of infection of the majority (≥ 95%) of human rabies cases globally [8].

There are currently sixteen classified lyssavirus species listed, with the virus abbreviation in parentheses: *Rabies lyssavirus* (RABV), *Lagos bat lyssavirus* (LBV), *Mokola lyssavirus* (MOKV), *Duvenhage lyssavirus* (DUVV), *European bat 1 lyssavirus* (EBLV-1), *European bat 2 lyssavirus* (EBLV-2), *Australian bat lyssavirus* (ABLV), *Aravan lyssavirus* (ARAV), *Khujand lyssavirus* (KHUV), *Irkut lyssavirus* (IRKV), *West Caucasian bat lyssavirus* (WCBV), *Shimoni bat lyssavirus* (SHIBV), *Bokeloh bat lyssavirus* (BBLV), *Ikoma lyssavirus* (IKOV) [9], *Lleida bat lyssavirus* (LLEBV) and *Gannoruwa bat lyssavirus* (GBLV) [9,10,11]. Recently, two related and recently discovered and unclassified lyssaviruses, i.e., Taiwan bat lyssavirus (TWBLV) and Kotalahti bat lyssavirus (KBLV), were isolated from bats [12,13,14]. To date, Africa has six lyssaviruses circulating: RABV, LBV, MOKV, DUVV, SHIBVand IKOV, with circulating RABV in terrestrial mammals remaining the most prominent lyssavirus accounting for the vast majority of human and animal rabies cases. The African RABVs have been sub-divided into four lineages: Africa 1a (restricted to North and West Africa), 1b (limited to South-East Africa), 2 (includes wild-type strains from several central and eastern African countries), 3 and 4 (does not group with any of the three African RABV lineages), based on phylogenetic analyses [15]. These lineages are separated geographically and have different host reservoirs. For instance, Africa 3 RABVs, previously known as “viverrid viruses” and now referred to as the “mongoose rabies biotype”, are predominantly confirmed in the yellow mongoose (*Cynictis penicillata*) in South Africa, but also from other wildlife carnivore species, such as the slender mongoose (*Galerella sanguinea*) and the African civet (*Civettictis civetta*) in Zimbabwe, raising questions of whether the African civet could be a maintenance host species of this biotype [16,17]. Historically, the mongoose rabies biotype, genetically distant from all dog RABV variants, was isolated on several occasions in the 1970s from the *Herpestidae* species, primarily the slender mongoose, and this provided some evidence that this host species could potentially be the reservoir host species for the mongoose RABV variant in Zimbabwe [18]. Subsequently, this mongoose variant was isolated from mustelids (such as the honey badger *Mellivora capensis*) and, increasingly, from the African civet in the 1990s [19], suggesting that there is no single definitive host reservoir for this variant. This observation is in contrast to that of the yellow mongoose *Cynictis penicillata*, clearly the definitive reservoir host for the mongoose rabies variant in South Africa [16]. More recently, a highly divergent lyssavirus, IKOV, was recovered from an African civet in Tanzania [20,21,22]. The continual identification of lyssaviruses from wildlife species such as the African civet raises additional questions as to whether and, if so, how the African civet is involved in the transmission and maintenance of lyssaviruses, or whether it is merely a dead-end host.

**Figure 1:**
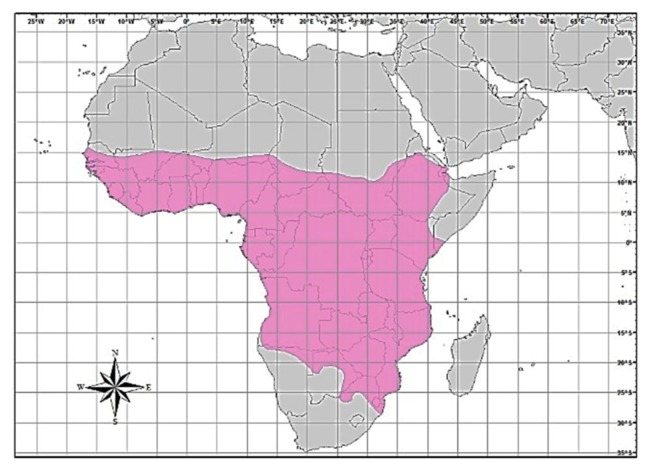
Map of Africa showing the distribution (brightly-coloured region) for the African civet [26].

In Zimbabwe, African civet rabies cases were generally diagnosed during jackal rabies outbreaks [23], suggesting a spill-over of infection. Furthermore, wildlife rabies contributes approximately 25% of all laboratory-confirmed rabies cases in Zimbabwe [24]. Of the wildlife rabies submitted for rabies testing between 2010 and 2017, jackals comprised 35.5% of positive cases, followed by 3.2% each for the African civet, lion, kudu and zebra (personal communication, Lambert Gwenhure, Central Veterinary Laboratory, Harare, Zimbabwe). The African civet is a common terrestrial viverrid species found in most of sub-Saharan Africa [25,26]. Its geographical distribution is confined to the African continent (south of the Sahara), but is also found throughout all other West-African countries, from Senegal right through to Ethiopia in the east. In the southern parts of the continent, civets are found in the extreme southern parts of Somalia, in Kenya, Uganda, the Democratic Republic of Congo (DRC), Angola, Tanzania, Zambia and Mozambique, extending to Botswana on the eastern border with Zimbabwe and the Limpopo province (in South Africa), Namibia and the whole of Zimbabwe (Figure 1).

The African civet is characterised by a wide head with a pointed muzzle, small eyes and small, rounded ears. It is a nocturnal, terrestrial and opportunistic mammal, and is the largest African member of the family *Viverridae*, which includes genets (small-spotted genet *Genetta genetta*) and large-spotted genets *Genetta tigrina* [26]. The African civet has a characteristic black streak across the face and a white patch on its forehead. As a way of intimidating an intruder, the African civet increases its size, exposing its back, and through this process limits physical contact with other carnivores. This wildlife carnivore is omnivorous and nocturnal, and is recognized for scavenging on cave floors for food, which could feasibly justify the concept that interactions with cave-dwelling bats could also occur with the added risk of viral cross-species transmission events [26].

In this study, all the available African civet lyssaviruses at Onderstepoort and the Animal and Plant Health Agency (AHPA, UK) were compared with those from other wildlife and domestic carnivore species, with the intention of identifying the lyssaviruses that are circulating in this unusual wildlife species and to determine whether these viruses are being maintained and transmitted by African civets, or if African civets are merely incidental hosts with no influence on the evolution of these complex viruses. These data highlight the importance of the molecular characterisation of confirmed rabies-positive cases, particularly for wildlife species such as African civets, but also known host reservoir species, such as the RABV biotype, as the lyssavirus species is not always as expected.

## 2. Materials and Methods 

### 2.1. Viruses

Viruses archived at both the Onderstepoort Veterinary Institute (OVI, Pretoria, South Africa) and the Animal and Plant Health Agency (APHA, Surrey, UK) were retrieved and included in this study. The epidemiological information of the viruses included in the investigation [South Africa (*n* = 11) and Zimbabwe (*n* = 12)] is presented in Table 1. Viruses recovered from host species before the year 2000 were passaged once in mice, freeze-dried and then stored at −20 °C, whereas for all specimens received post-2000, the original lyssavirus-infected brain tissues were stored frozen (at −80 °C).

### 2.2. Viral RNA Extractions, Reverse-Transcription PCR and Sequencing

Total viral RNA extractions were performed with Tri Reagent (Product #T9424, Sigma, St. Louis, MO, U.S.A) according to the manufacturer’s guidelines. Complementary DNA (cDNA) was synthesised using Superscript III reverse transcriptase (Invitrogen, Renfrew, UK) on approximately 1 µg of total RNA in a total volume of 20 µl as per the manufacturer’s instructions, using 2 pmol of JW12 and GT1Mfor2 primers for amplification of the N and G genes, respectively. Two microlitres of RNAse H were added to the reaction mixes after cDNA synthesis to remove the RNA and incubated for a further 20 min at 37 °C. The PCR reaction mixes for amplification of the two genomic regions were then assembled using the JW12 and 304R primers to amplify the full nucleoprotein gene (N) of the virus and the GT1Mfor2 and GT1 N-L rev or VIVMF and VIVLR primer pairs for the G gene, depending on whether the virus was a canid or mongoose RABV variant (Table S1 for primer details, [27,28,29]). One microlitre of cDNA was mixed with 5 µl of 10X buffer, 3 µl of 25 mM MgSO4, 1 µl of 2 mM dNTPs, 15 pmol of each primer and 1 U KOD enzyme (Merck Millipore, Edinburgh, UK). Fifty microlitres of PCR reactions were run on a 2720 thermal cycler (Applied Biosystems, Foster City, CA, USA) on the following PCR program: 95 °C for 2 min, then 35 cycles of 95 °C for 20 sec, 55 °C for 20 sec, 70 °C for 30 sec, and a final extension at 70 °C for 10 min. PCR products were then analyzed on 1.8% agarose gels and visualized by SYBR safe (Invitrogen) DNA gel-staining. The PCR amplicons were purified using spin-columns (Qiagen, Hilden, Germany) and subjected to sequencing directly (primers available on request), using Big Dye V3.1 sequencing kit (Applied Biosystems) on an ABI3100 machine. Representative historic and contemporary complete N sequences of putative dog-related variants independently established in black-backed jackals and bat-eared foxes, as well dog-maintained lineages, circulating in South Africa were included in the analysis. Conserved specific amino acid signatures (exclusively found in African civets) in the rabies virus isolates originating from African civets (but not in rabid dogs) are consistent over time and may be a good marker correlating with the establishment of formerly dog-maintained RABVs in the African civet population, as was observed in striped skunks (*Mephitis mephitis*) relative to fox and raccoon rabies in North America

### 2.3. Phylogenies and Evolutionary Analysis

**Figure 2:**
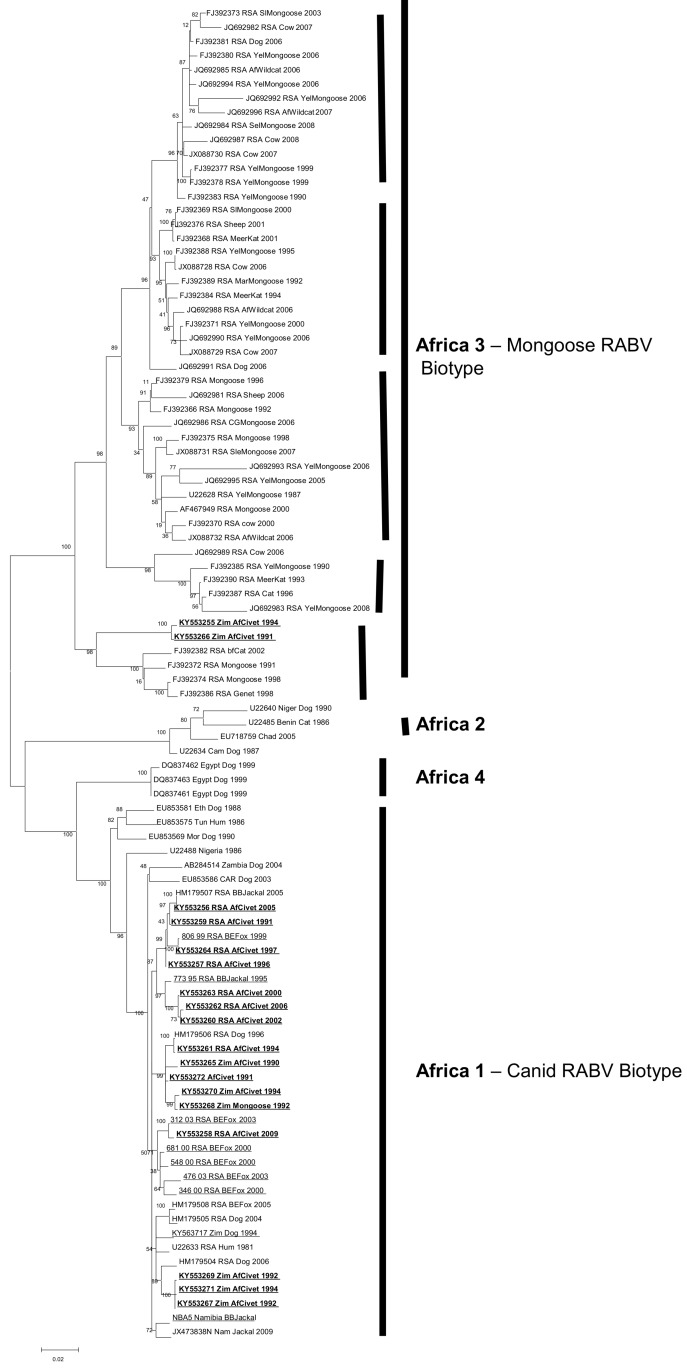
Maximum likelihood phylogenetic tree of complete N-gene coding sequence of representative African canid rabies virus (RABV) sequences including the African civet N-gene sequences (bold and underlined). African RABV lineages are highlighted by solid lines. The five clades within Africa 3 are also indicated by bold and vertical lines.

**Figure 3:**
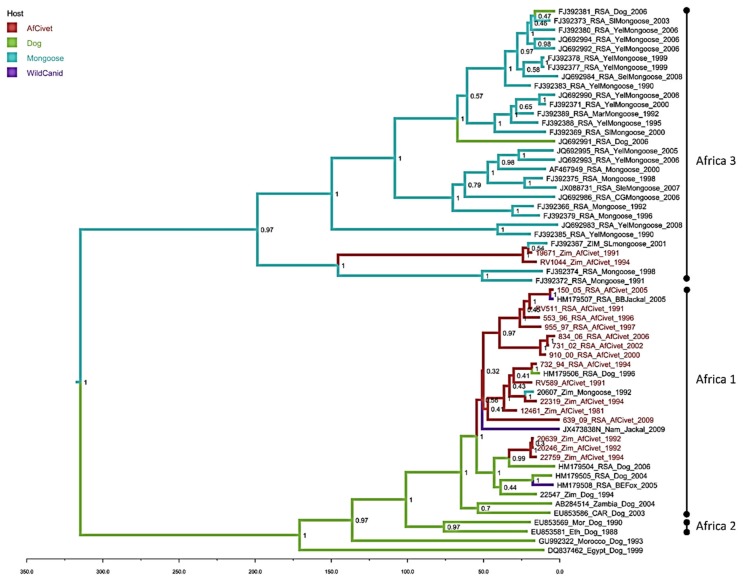
Maximum clade credibility tree from Bayesian reconstruction of 59 rabies virus full nucleoprotein sequences (1353nt), using an HKY +G+I model of evolution with a strict molecular clock and constant population prior, for 30 million iterations in BEAST (v1.8.1). The maximum clade credibility tree was chosen using TreeAnnotator (v1.8.0) and visualised using FigTree (v1.4.0). African civet sequences are highlighted in red, posterior support is given at key nodes, and branches are coloured by the host with the highest probability at the ancestral node.

Maximum likelihood phylogenetic reconstructions of the complete N gene civet sequences were performed in MEGA 6 using 1000 bootstrap replicates [30]. A GTR nucleotide substitution model with rate variation and a proportion of invariant sites (Gamma+I) was determined to best fit the data using Akaike Information Criterion in MEGA 6 (Figure 2 and Figure 3). To further investigate the genetic relationships of the African civet viruses relative to RABV lineages circulating in Africa, sequences of the complete nucleoprotein gene (N-gene – 1353 bp) were analysed with other complete N-gene sequences available in Genbank (see supplementary file, Table S2). Sequences derived in this study (*n* = 23) were compared with other complete N-gene sequences available in Genbank (*n* = 59). A Bayesian Markov Chain Monte Carlo simulation was also used to infer phylogenetic relationships (BEAST packagev1.8.1) [31]. An HKY nucleotide substitution model with rate variation and a proportion of invariant sites (Gamma+I) was used. Relaxed and strict molecular clock models, and either constant or Bayesian skyline population priors were used in combination and compared using a modified Akaike information criterion (AICM) in Tracer v1.5, as described previously [32]. To give a visual representation of the inferred ancestral host for each clade, each sequence was given a discrete trait corresponding to the species in which the virus sequences were detected. All likely ‘dead-end’ hosts (unable to further transmit rabies virus infection) were removed and the remaining hosts were grouped into mongoose, African civet, canine canid (domestic) and wild canid categories. A chain length of 30 million iterations, sampling every 3000 states, gave effective sample sizes of over 100. Maximum clade credibility trees were annotated using TreeAnnotator (v1.8.1) after 10% of trees were discarded, visualised with FigTree (v1.4.0) and coloured by the host species with the highest posterior probability (Figure 3).

## 3. Results

### RT-PCR, Sequencing and Phylogenetic Reconstruction

Full coding N-gene sequences (1353nt) were analysed with previously published N-gene sequences representing the RABV diversity across Africa. Partial G-gene sequences (600nt) were also generated for the African civet viruses. The phylogenetic trees generated from the G-gene sequences were topologically similar to the N-gene phylogenies; therefore, only the N-gene dataset (Table 1) was used in subsequent analyses due to the larger dataset of related African RABV N-gene sequences publicly available (Table S2). Four distinct lineages were delineated; Africa 1, Africa 2 and Africa 4 (all canid RABV biotype), and Africa 3 (mongoose RABV biotype) (see Figure 2). All the civet viruses were exclusively placed in Africa 1 and 3 lineages. 

The mongoose rabies biotype (Africa 3) was delineated into five previously recognized clades, four of which were spatially dispersed throughout South Africa (the smallest clade representing viruses from Mpumalanga province of South Africa, the second one representing viruses recovered from both the northern parts of the Free State and North-West provinces; the third one is the major clade comprising the northern and southern parts of the Northern Cape as well as the Eastern Cape provinces, and the last one from exclusively the Northern Cape Province). The fifth clade (#5) consists of rabies viruses originating from wildlife host species, including two African civet samples and a slender mongoose, spanning the north east to the south west of Zimbabwe [16], Figure 2. 

The African civet RABV (within the Africa 1 lineage) have significantly lower bootstrap support values than those in the Africa 3 lineage. The three sub-clusters observed in Africa 1 consist of a mixture of viruses from both Zimbabwe and South Africa with the exception of one of a subclade, which exclusively contains rabies viruses from African civets and a black-backed jackal collected in South Africa. 

The Bayesian phylogenetic analysis (Figure 3) with sequences exclusively from dogs and wildlife has a similar topology to the maximum likelihood tree (Figure 2), supporting the division of the African civet sequences into the previously established Africa 1 (canid biotype) and Africa 3 (mongoose biotype) clades (Figure 3). The majority of represented civet sequences are in the Africa 1, the canid biotype clade and these data support a common ancestor of these civet nucleotide sequences, with the represented dog sequences, that occurred within the last 50 years. There are three well-supported subclades (HPD >90%) in Africa 1; one contains almost exclusively RSA civet sequences from 1991–2005, grouping with one sequence from a black-backed jackal. A second subclade contains four civet sequences from both Zimbabwe and RSA, with single dog and mongoose samples. A third small cluster of three civet rabies viruses sequences from Zimbabwe 1992–1994 has a later common ancestor, within the last 25 years. There are only two civet sequences clustering with the Africa 3 mongoose biotype and, despite being isolated in 1991 and 1994, they are most closely related to a mongoose sequence from 2001.

## 4. Discussion

Cross-species transmission (CST) events occur when a pathogen (normally a virus) in a specific reservoir animal is transmitted to another host. Such CST events rarely result in successful propagation within a new host, as exemplified by lyssaviruses [33]. In these previous studies, it was shown that raccoons were four times more likely to transmit the rabies virus to other species than skunks. In Southern Africa, the spill-over of the mongoose rabies biotype to canid hosts (such as the domestic dog), apparently do not establish secondary dog-to-dog transmissions and result in dead-end infections [34]. Data from our current study demonstrated and revealed CST events between dog rabies virus maintenance hosts (namely dogs and jackal species) and the African civet on the one hand, and the African civet and other *Herpestidae* species (slender mongoose), particularly in those geographical areas where host ranges of these species interface, on the other hand. Host and environmental factors that facilitate CST are, to a large degree, unknown [35]. RABV illustrates one of those RNA-containing multi-host pathogens that circulates in a diverse host reservoir, highlighting its ability to opportunistically infect multiple hosts [36]. Transmission of RABVs is heavily influenced by host ecology and opportunities for contact between susceptible species.

Using complete nucleotide sequences of the N gene of the African civet lyssaviruses in our archive, together with other previously sequenced African civet viruses, comprehensive genetic analyses were undertaken. These data support a clear and deeply rooted ancestral division of the represented RABVs into the well-described RABV biotypes from southern Africa: canid and mongoose. Historically, the mongoose RABV sequences from the African civet demonstrated a nucleotide sequence divergence of between 1.3%–15.4% over the genomic region studied, displaying a larger genetic diversity compared to African civet sequences (of 15.2%) in the Africa 1 clade [16]. A previous study demonstrated that virus genetic diversity is higher in heterologous passages compared to homologous passages [37], at least in the case of the passage of a dog-adapted virus into foxes. Presumably due to a lower contact risk with humans, and possible reduced neurovirulence and pathogenicity compared to the canid RABVs, fewer human cases are attributed to mongoose rabies and it is, therefore, considered less of a public health threat [38,39,40]. Findings from studies by Seo et al. demonstrated pathogenicity differences of South African canid and mongoose RABVs in a murine model by assessing clinical signs and survivorship. The C-termini of the glycoprotein of the South African canid and mongoose RABVs showed that the canid rabies virus exhibited a phenotype associated with survival in their murine model, which was not found in the mongoose variant. These observations confirmed that, indeed, the canid rabies variant is more virulent than the mongoose rabies variant under experimental conditions in a murine model. In contrast, the canid RABV variant has been shown to be more opportunistic, infecting both domestic and wildlife host species. There are fewer host species infected with the mongoose rabies biotype in the Africa 3 RABV clade, with the majority being bona fide mongooses, in comparison to the Africa 1 clade, albeit subject to sampling bias. However, there are two RABV sequences from dogs within the Africa 3 (mongoose rabies biotype) clade, demonstrating that spill-over of the mongoose rabies biotype into other species does occur. The majority of the RABV sequences isolated from civets are found in the Africa 1 (canid biotype) RABV clade. These civet sequences are further divided into two subclades, one with dates of origin ranging from 1981 to 2009 and locations including RSA and Zimbabwe, the other restricted to three sequences from Zimbabwe in the early 1990s. These inferred evolutionary relationships raise the possibility of maintenance of RABV within civet populations for up to 50 years, with recent spill-over into other wild canids and dogs [41]. The tree topology not only indicates that the African civets have been exposed to dog-maintained RABVs for as long as the dog variant has been maintained in this species in this region, but also that this long term exposure could have led to the establishment of originally dog-maintained RABV in African civet populations.

Sequences of putative dog-related variants independently established in black-backed jackals and bat-eared foxes, as well as dog-maintained lineages circulating in southern Africa with conserved specific amino acid signatures otherwise also found in rabid civets, may demonstrate a marker for the establishment of formerly dog-maintained RABVs in the African civet population [42]. This civet maintenance host was not previously considered, likely due to the opportunistic and solitary nature of African civets [17,22]. However, sampling bias has a significant effect on these and other evolutionary analyses, as we cannot rule out the possibility of RABV maintenance in other host species that are not included in these data. For example, the relative lack of dog or jackal sequences collected from similar locations and dates to the civet samples prevents definitive conclusions to be drawn.

Surveillance data from southern Africa have demonstrated that wildlife rabies is a significant veterinary and public health problem, specifically with RABVs readily crossing the species barriers partly due to the overlapping host ranges (sympatry) of a susceptible species with that of a species already maintaining a cycle of RABV [24,43]. The known home range of the African civet interfaces with that of the slender mongoose in Zimbabwe and, hence, encourages the likely exchange of viruses between these host species. Whereas common and similar cellular and immunological traits are key to initial infection of a new host, recipient host population ecology is equally pivotal. From the available data from observations and studies undertaken in other parts of Africa, the African civet appears to be susceptible to infection with a variety of lyssaviruses, namely the canid and mongoose in southern Africa and, more recently, with IKOV in Tanzania [17,20]. The infrequent detection of lyssaviruses such as IKOV may point to the fact that African civets are indeed incidental hosts of this lyssavirus, but, alternatively, this may be due to low surveillance activities. The incubation periods of RABV infection in African civets in captivity are variable [25]. For instance, in Zimbabwe, an African civet was linked to an unusually long incubation period of at least 186 days compared to 11 days in a similar species in Nigeria [44], but this could be due to strain differences. Animals with prolonged incubation periods of rabies infection are believed to act as effective reservoirs of disease, bridging infection over periods where the propagation in endemic hosts is not logically favourable, for instance, but can contribute to the maintenance of the disease between rabies epidemics in jackals, harbouring the infection whilst the jackal population density recovered.

In Zimbabwe, the African civet is not considered a major host for RABV (it only accounts for about 1.5% of all confirmed wildlife cases), and the epidemiology suggests that most of these cases are detected during epidemics of jackal rabies. Initial monoclonal antibody typing studies of African civet viruses [18,41,45] showed that they were indistinguishable from jackal viruses (Africa 1, canid rabies biotype), but more recent data supported the notion that African civet populations may be capable of supporting the maintenance of enzootic mongoose rabies [17]. Although Haydon 2012 discussed and highlighted the challenges of identifying reservoirs of RABV infection, Lembo et al. (2008) [46] demonstrated that persistence of infection in a population is a prerequisite for identifying reservoirs. Considering the trends analyses (2000–2015) in South Africa, rabies in the African civet occurred sporadically and represented a mere 0.3% of the total confirmed positive rabies cases. These data are heavily skewed due to sampling bias. African civets are, incidentally, not found close to human habitats; therefore, lyssavirus-positive African civet cases are likely to be underrepresented. However, African civets seem to be highly susceptible to a more divergent plethora of lyssaviruses, which include dog-mediated and dog-related RABV and IKOV. Whether the African civet is only involved in a multispecies cycling of the infection, similar to that shown in striped skunks (*Mephitis mephitis*) and in fox and raccoon rabies in North America, or can maintain its own enzootic cycle of RABV, is uncertain when analysing epidemiological data alone. Indeed, the data presented here suggest that African civets may be an important wildlife species in the maintenance of lyssaviruses, particularly the African RABV biotypes. Indeed, RABV survives favourably in the wild because it can infect a large spectrum of animals, thereby maximising its replication and dispersal opportunities [47]. The findings from this study highlight the importance of sequencing confirmed rabies-positive cases, particularly for wildlife species such as African civets, but also known host reservoirs species, such as the RABV biotype, as the lyssavirus species does not always behave as expected. Further analysis of African civet rabies-positive cases and other poorly understood wildlife species and improved surveillance of rabies cases from the same regions will help unravel the complexities of lyssavirus epidemiology.

## 5. Conclusions

This study has shown that African civets are infected by both canid and mongoose RABV biotypes and occasionally by other lyssaviruses, such as IKOV. There is, however, no evidence of rabies causing population declines in African civets. The data may suggest that maintenance of RABV within African civets occurs and that the African civet should be considered a possible host reservoir in southern Africa. These data reinforce the notion that it is important that the typing of viruses from wildlife species is part of a routine surveillance where such facilities exist, or through collaboration with rabies reference laboratories to undertake genetic typing. The identification of canid and mongoose rabies viruses do indeed demonstrate the extent that the African civet interacts with other domestic and wildlife host species in southern Africa.

## Figures and Tables

**Table 1:** Details of all known RABV positive African civets both from this study and historically, including details of additional relevant RABV positive samples obtained during the study.

Original ID	Host	Date	Locality and Country of Origin	GenBank N Gene	Africa Lineage (1, 2 or 3)
19671	African civet	10.08.1991	Rusape, Zimbabwe	KY553266	3
20246	African civet	15.01.1992	Macheke, Zimbabwe	KY553267	1
20639	African civet	23.04.1992	Macheke, Zimbabwe	KY553269	1
21179	African civet	11.11.1992	Penhalonga, Zimbabwe		3
22319	African civet	30.06.1994	Concession, Zimbabwe	KY553270	1
22574	African civet	1994	Wedza, Zimbabwe	KY553255	3
23639	African civet	18.09.1995	Juliusdale, Zimbabwe		nd
22759	African civet	16.12.1994	Shamva, Zimbabwe	KY553271	1
27988	African civet	15.03.2001	Chiredzi, Zimbabwe		3
18959	African civet	25.09.1990	Kadoma, Zimbabwe	KY553265	nd
28593	African civet	29.05.2003	Rugoti, Zimbabwe		3
28841	African civet	2002	Mutoko, Zimbabwe		3
0698/91	African civet	1991	Limpopo, South Africa	KY553259	1
0732/94	African civet	1994	Ellisras, South Africa	KY553261	1
0553/96	African civet	1996	Ellisras, South Africa	KY553257	1
0955/97	African civet	1997	Soutpansberg, South Africa	KY553264	1
0354/98	African civet	1998	Limpopo, South Africa		nd
0396/00	African civet	2000	Bloemfontein, South Africa		nd
0910/00	African civet	2000	Ellisras, South Africa	KY553263	1
0731/02	African civet	2002	Thabazimbi, South Africa	KY553260	1
0150/05	African civet	2005	Ellisras, South Africa	KY553256	1
0834/06	African civet	2006	Ellisras, South Africa	KY553262	1
0639/09	African civet	2009	Gordonia, South Africa	KY553258	1
20607	Mongoose	1992	Karoi, Zimbabwe	KY553268	1
RV589	Honey Badger	1991	Zimbabwe	KY553272	1
22547	Dog	1994	Zimbabwe	KY563718	1
806/99	Bat-eared fox	1999	South Africa	DQ489796	2iii
548/00	Bat-eared fox	2000	South Africa	DQ489807	1
681/00	Bat-eared fox	2000	South Africa	DQ489844	2iv
346/00	Bat-eared fox	2000	South Africa	DQ489814	2ii
312/03	Bat-eared fox	2003	South Africa	DQ489826	2i
476/03	Bat-eared fox	2003	South Africa	DQ489828	2ii
NBA5	Black-backed jackal		Namibia, Etosha	DQ194887	2iv
773/95	Black-backed jackal	1995	South Africa	DQ489861	2iii

## Supplementary Materials

The following are available online at https://www.mdpi.com/1999-4915/12/4/368/s1, Table S1: PCR primers used in the study, Table S2: RABV sequences used for phylogenetic analysis with African civet sequences (Figure 2).
