# Supplementary material for: Rabies in the African Civet: An Incidental Host for Lyssaviruses?
Source: Viruses. 2020 Mar 27;12(4):368. doi: 10.3390/v12040368 (PMC7232503; doi:10.3390/v12040368)
Supplement: Supplementary file 1 [file viruses-12-00368-s001.zip › suppl/viruses-596058.Table S1.pdf]

Table S1: PCR primers used in the study

Primer name	Primer location (PV)	Gene amplified	Primer sequence (5'-3')	Publication
JW12	55-73	N	ATGTAACACCYCTACAATGG	Heaton et al., 1997
304R	1514-1533	N	TTGACGAAGATCTTGCTCAT	McElhinney et al., 2011
GT1 MFor2	3286-3305	G	CTATTAACATCCCTCAAAAG	This study
GT1 N-L Rev	5966-5986	G	TCCCAGTCTAGGGCRTTCATG	
VivMF	3081-3099	G	GATTCCTCTCTGCTTCTAG	Van Zyl N. et al., 2010
VivLR	5521-5543	G	CAAAGGAGAGTTGAGATTGTAGT	
