# Supplementary material for: Rabies in the African Civet: An Incidental Host for Lyssaviruses?
Source: Viruses. 2020 Mar 27;12(4):368. doi: 10.3390/v12040368 (PMC7232503; doi:10.3390/v12040368)
Supplement: Supplementary file 1 [file viruses-12-00368-s001.zip › suppl/viruses-596058.Table S2.pdf]

Table S2 RABV sequences used for phylogenetic analysis with African civet sequences (Figure 2). All are complete N gene sequences unless indicated otherwise. Sequences used in Figure 3 Bayesian host trait analysis are underlined.

GenBank ID	Original ID	Country	Host	Year Isolated	RABV Variant
AB284514	ZAMRAV23/04	Zambia	Dog	2004	Africa 1 Canid
AF467949	710/90	RSA	*Cynictis penicillata*	1990	Africa 3 Mongoose
DQ837461	S1-81	Egypt	Dog	1999	Africa 4
DQ837462	S2-81	Egypt	Dog	1999	Africa 4
DQ837463	S3-81	Egypt	Dog	1999	Africa 4
EU718759	Chad_71_164	Chad	Dog	2005	Africa 2
EU853569	9016MAR	Morocco	Dog	1990	Africa 2
EU835375		Tunisia	Human	1986	Africa 2
EU853581	8808ETH	Ethiopia	Dog	1988	Africa 1 Canid
EU853586	07128RCA	CAR	Dog	2003	Africa 2
FJ392366	19-92	RSA	Mongoose	1992	Africa 3 Mongoose
FJ392367	22107	Zim	Slender Mongoose	2001	Africa 3 Mongoose
FJ392368	23-01	RSA	Meerkat	2001	Africa 3 Mongoose
FJ392369	28-00	RSA	Slender Mongoose	2000	Africa 3 Mongoose
FJ392370	30-00	RSA	Bovine	2000	Africa 3

					Mongoose
FJ392371	32-02	RSA	Yellow Mongoose	2002	Africa 3 Mongoose
FJ392372	113-91	RSA	*Atilux paludinosus*	1991	Africa 3 Mongoose
FJ392373	155-03	RSA	Slender Mongoose	2003	Africa 3 Mongoose
FJ392374	211-98	RSA	Canine	1998	Africa 3 Mongoose
FJ392375	221-98	RSA	*Suricata suricatta*	1998	Africa 3 Mongoose
FJ392376	228-01	RSA	Sheep	2001	Africa 3 Mongoose
FJ392377	279-99	RSA	Yellow Mongoose	1999	Africa 3 Mongoose
FJ392378	344-99	RSA	Yellow Mongoose	1999	Africa 3 Mongoose
FJ392379	364-96	RSA	Mongoose	1996	Africa 3 Mongoose
FJ392380	381-06	RSA	Yellow Mongoose	2006	Africa 3 Mongoose
FJ392381	385-06	RSA	Dog	2006	Africa 3 Mongoose
FJ392382	389-02	RSA	Black Footed Cat	2002	Africa 3 Mongoose
FJ392383	420-90	RSA	Yellow Mongoose	1990	Africa 3 Mongoose
FJ392384	485-94	RSA	*Suricata suricatta*	1994	Africa 3 Mongoose

FJ392385	669-90	RSA	Yellow Mongoose	1990	Africa 3 Mongoose
FJ392386	718-98	RSA	*Genetta genetta*	1998	Africa 3 Mongoose
FJ392387	759-96	RSA	Cat	1996	Africa 3 Mongoose
FJ392388	767-95	RSA	Yellow Mongoose	1995	Africa 3 Mongoose
FJ392389	878-92	RSA	Mar Mongoose	1992	Africa 3 Mongoose
FJ392390	926-93	RSA	*Suricata suricatta*	1993	Africa 3 Mongoose
GU992322	93128MAR	Morocco	Dog	1993	Africa 2
HM179504	262-06	RSA	Dog	2006	Africa 1 Canid
HM179505	567-04	RSA	Dog	2004	Africa 1 Canid
HM179506	479-96	RSA	Dog	1996	Africa 1 Canid
HM179507	819-05	RSA	Black-backed Jackal	2005	Africa 1 Canid
HM179508	31-05	RSA	Bat Eared Fox	2005	Africa 1 Canid
JQ692981	06-06	RSA	Sheep	2006	Africa 3 Mongoose
JQ692982	06-07	RSA	Cow	2007	Africa 3 Mongoose
JQ692983	116-08	RSA	Yellow Mongoose	2008	Africa 3

					Mongoose
JQ692984	131-08	RSA	Slender Mongoose	2008	Africa 3 Mongoose
JQ692985	18-06	RSA	African Wildcat	2006	Africa 3 Mongoose
JQ692986	200-06	RSA	*Garelella pulverulenta*	2006	Africa 3 Mongoose
JQ692987	27-08	RSA	Bovine	2008	Africa 3 Mongoose
JQ692988	456-06	RSA	African Wildcat	2006	Africa 3 Mongoose
JQ692989	50-06	RSA	Bovine	2006	Africa 3 Mongoose
JQ692990	57-06	RSA	Yellow Mongoose	2006	Africa 3 Mongoose
JQ692991	956-06	RSA	Dog	2006	Africa 3 Mongoose
JQ692992	265-06	RSA	Yellow Mongoose	2006	Africa 3 Mongoose
JQ692993	418-06	RSA	Yellow Mongoose	2006	Africa 3 Mongoose
JQ692994	696-06	RSA	Yellow Mongoose	2006	Africa 3 Mongoose
JQ692995	257-05	RSA	Yellow Mongoose	2005	Africa 3 Mongoose
JQ692996	13-07	RSA	African Wildcat	2007	Africa 3 Mongoose
JX088728	28-06	RSA	Bovine	2006	Africa 3 Mongoose

JX088729	235-07	RSA	Bovine	2007	Africa 3 Mongoose
JX088730	397-07	RSA	Bovine	2007	Africa 3 Mongoose
JX088731	416-07	RSA	Slender Mongoose	2007	Mongoose
JX088732	584-06	RSA	African Wildcat	2006	Africa 3 Mongoose
JX473838	178J09	Namibia	Jackal	2009	Africa 1 Canid
KY553268<br>KY553279 (G)<br>AY605016 (GL)	20607	Zimbabwe	Mongoose	1992	Africa 1 Canid
KY553272	RV589	Zimbabwe	Honey Badger	1991	Africa 1
KY563717<br>KY563715 G<br>AF177070 (GL)	22547	Zimbabwe	Dog	1994	Africa 1 Canid
U22488	8670NGA	Nigeria	Human	1983	Africa 1b
U22485	8697BEN	Benin	Cat	1986	Africa 2
U22628	1500AFS	RSA	Yellow mongoose	1987	Africa 3
U22633	8721AFS	RSA	human	1981	Africa 1b
U22634	8801CAM	Cameroon	Dog	1987	Africa 2
U22640	9012NIG	Niger	Dog	1990	Africa 2
This study genome<br>DQ489796 (N)<br>DQ431318 (GL)	806/99<br>(RV1435)	RSA	Bat-eared Fox	1999	Africa 1 Clade 2iii
This study	548/00	RSA	Bat-eared Fox	2000	Africa 1

genome<br>DQ489807 (N)<br>DQ431368 (GL)	(RV1448)				Clade 1
This study<br>genome<br>DQ489810 (N)<br>DQ431353 (GL)	681/00<br>(RV1451)	RSA	Bat-eared Fox	2000	Africa 1<br>Clade 2iv
This study<br>genome<br>DQ489814 (N)<br>DQ431282 (GL)	346/00<br>(RV1455)	RSA	Bat-eared Fox	2000	Africa 1<br>Clade 2ii
This study<br>genome<br>DQ489826 (N)<br>DQ431308 (GL)	312/03<br>(RV1476)	RSA	Bat-eared Fox	2003	Africa 1<br>Clade 2i
This study<br>genome<br>DQ489828 (N)<br>DQ431340 (GL)	476/03<br>(RV1480)	RSA	Bat-eared Fox	2003	Africa 1<br>Clade 2ii
This study<br>genome<br>DQ194887 (N)<br>DQ431325 (GL)	NBA5<br>(RV1829)	Namibia	Black-backed Jackal	2000	Africa 1<br>Clade 2iv
This study<br>genome<br>DQ194861 (N)<br>DQ431316 (GL)	773/95<br>(RV1869)	RSA	Black-backed Jackal	1995	Africa 1<br>Clade 2iii
